# Genetic Factors Associated With Nodulation and Nitrogen Derived From Atmosphere in a Middle American Common Bean Panel

**DOI:** 10.3389/fpls.2020.576078

**Published:** 2020-12-15

**Authors:** Atena Oladzad, Abiezer González, Raul Macchiavelli, Consuelo Estevez de Jensen, James Beaver, Tim Porch, Phillip McClean

**Affiliations:** ^1^Department of Plant Sciences, North Dakota State University, Fargo, ND, United States; ^2^Department of Agroenvironmental Sciences, University of Puerto Rico, Mayagüez, Puerto Rico; ^3^USDA-ARS, Tropical Agriculture Research Station, Mayagüez, Puerto Rico

**Keywords:** Phaseolus vulgaris, Rab GTPase, biological nitrogen fixation pathway, NRT1 genes, MATE efflux proteins, genome-wide association study, Aux/IAA family

## Abstract

Among grain legume crops, common beans (*Phaseolus vulgaris* L.) are considered to have poor biological nitrogen (N_2_) fixation (BNF) capabilities although variation in N_2_ fixing capabilities exists within the species. The availability of genetic panel varying in BNF capacity and a large-scale single nucleotide polymorphism (SNP) data set for common bean provided an opportunity to discover genetic factors associated with N_2_ fixation among genotypes in the Middle American gene pool. Using nodulation and percentage of N_2_-derived from atmosphere (%NDFA) data collected from field trials, at least 11 genotypes with higher levels of BNF capacity were identified. Genome-wide association studies (GWASs) detected both major and minor effects that control these traits. A major nodulation interval at Pv06:28.0–28.27 Mbp was discovered. In this interval, the peak SNP was located within a small GTPase that positively regulates cellular polarity and growth of root hair tips. Located 20 kb upstream of this peak SNP is an auxin-responsive factor AUX/indole acetic auxin (IAA)-related gene involved in auxin transportation during root nodulation. For %NDFA, nitrate (NO_3_^−^) transporters, *NRT1:2* and *NRT1.7* (Pv02:8.64), squamosa promoter binding transcriptome factor (Pv08:28.42), and multi-antimicrobial extrusion protein (MATE) efflux family protein (Pv06:10.91) were identified as candidate genes. Three additional QTLs were identified on chromosomes Pv03:5.24, Pv09:25.89, and Pv11: 32.89 Mbp. These key candidate genes from both traits were integrated with previous results on N_2_ fixation to describe a BNF pathway.

## Introduction

Common bean (*Phaseolus vulgaris* L.) is a major crop of smallholder farmers in Latin America, the Caribbean, and Eastern and Southern Africa. In these regions, common beans are often grown on soils deficient in nitrogen (N_2_; Fenta et al., 2020) and among grain legumes, the species has a relatively low biological N_2_ fixation (BNF) capacity (Mnasri et al., 2007). The application of N_2_ fertilizer can increase bean yields, but the input is often too expensive and/or unavailable to smallholder farmers.

Biological N_2_ fixation is a process in which rhizobia microorganisms in the soil convert atmospheric N_2_ to ammonia (NH_3_). This process generates a usable form of nitrogenous compounds for the plant in an economical and environmentally friendly manner through nodulation (Akter et al., 2018). Nodulation is a symbiotic interaction between rhizobia and the legume plant in which the plant provides carbohydrate to the rhizobial species, and the bacteria convert atmospheric N_2_ into ammonium for use by the plant (Wang et al., 2018). Although common bean is considered a poor N fixer, genetic diversity for N_2_ fixation exists within the species. This diversity enables the selection of germplasm with enhanced BNF capacity (Buttery et al., 1987; Barron et al., 1999; Farid et al., 2017; Kamfwa et al., 2019). Pereira et al. (1993) were able to significantly increase the nodule number of common bean lines after three cycles of recurrent selection in a controlled environment. The results demonstrated that nodule number is a heritable trait, and Bliss (1993) suggested that nodule number could be used as a criterion to estimate BNF capacity.

The identification of key genetic factors associated with nodulation and the physiological response in a diverse collection of germplasm may enable the development of molecular markers to select for enhanced levels of N fixation capacity. Previously, genetic mapping (Nodari et al., 1993; Heilig et al., 2017; Kamfwa et al., 2019) and transcriptional regulation studies of the BNF response (Blanco et al., 2009) provided valuable information regarding BNF in common bean. However, few genetic studies to understand the genetic regulation of BNF across a diverse panel of common bean germplasm have been conducted.

Selecting bean lines with greater BNF capacity in breeding nurseries with high N_2_ fertilizer soil amendments negates the plant’s need for effective BNF capacity. Although quantifying BNF itself is difficult, studying related traits, such as seed yield, photosynthesis, biomass partitioning, root morphology, plasticity, and nodulation, along with environmental effects, can provide a framework for the genetic improvement of BNF in common bean (Diaz et al., 2017; Fenta et al., 2020).

Initially, the lack of a simple, rapid, and economical method to measure BNF capacity limited genetic improvement (Purcell, 2009). The discovery that BNF capacity in common bean is related to root nodule number and weight (Pereira et al., 1993), enabled the development of an effective screening procedure. BNF capacity can also be indirectly quantified by calculating percentage of N_2_-derived from atmosphere (%NDFA). This procedure compares the amount of ^15^N (heavy N_2_ isotope) for a genotype relative to the ^15^N value of a control genotype that does not have the capacity to nodulate and fix N_2_. This value, known as *δ*^15^N, is obtained from the ratio of ^15^N: ^14^N. The abundance of ^15^N in different parts of a legume plant can be obtained by calculating the relative deviation from the ^15^N: ^14^N ratio. In a recent study, the significant genetic variation among recombinant inbred lines (RILs) derived from Middle American genotypes for %NDFA demonstrated that the genetic variation among RILs would be mostly additive (Farid et al., 2017). Furthermore, significant QTL for %NDFA was detected within Andean gene pool (Ramaekers et al., 2013). These significant and repeatable differences between lines in both common bean gene pools suggest additive genetic effects play important role for %NDFA. Previous studies in soybean (*Glycine max* L. Merr.) confirmed that the differences in *δ*^15^N between nodulated roots from different lines or tissues are a robust means for quantifying %NDFA (Wanek and Arndt, 2002). Polania et al. (2016) noted that the natural abundance of ^15^N estimated using seed tissue would be an appropriate estimate for BNF capacity of common bean.

Given the importance of the nodule numbers and %NDFA in BNF estimation, genetic mapping these two traits may lead to a better understanding of the genetic factors affecting BNF capacity in common bean. In this study, we evaluated a panel specifically developed for abiotic stress tolerance. The aim of this study was to: (1) characterize common bean genotypes with high capacity for N_2_-fixation in tropical soil conditions and (2) discover the genomic regions and candidate genes associated with nodulation and %NDFA under tropical soil conditions.

## Materials and Methods

Bean Abiotic Stress Evaluation (BASE_120) field trials (Oladzad et al., 2019) were conducted at the Isabela Substation of the Agricultural Experiment Station of the University of Puerto Rico. The substation is located on the northwestern coastal plain of Puerto Rico at 18.468 N, −67.042 W at an altitude of 128 m above the sea level. The average annual minimum/maximum temperature at the Isabela Experimental Substation is 22.2/27.8°C, with an average annual rainfall of 1,630 mm. The trial was conducted in a field with Coto Clay soil, a very-fine, kaolinitic, isohyperthermic Typic Eutrustox. Soil subsamples were taken at random from different positions in the fields, where the BASE_120 trials were conducted. The subsamples were mixed to prepare a composite sample for each field. The soil analyses were conducted at AgSource Harris Laboratories in Lincoln, NE (Supplementary Table S1).

Field trials were planted in a randomized complete block design with five replications in four growing seasons; June and November 2015, June 2016, and June 2018. The experimental units were single 3-m rows with 0.76-m spacing between rows. Each BASE_120 trial included 118 common bean and two tepary bean (*Phaseolus acutifolius* L.) genotypes (Supplementary Table S2). Entries in the BASE_120 trials included elite bean breeding lines and cultivars from Zamorano University in Honduras, the International Center for Tropical Agriculture (CIAT) in Colombia, the University of Puerto Rico, USDA-ARS Tropical Agriculture Research Station in Puerto Rico, and Michigan State University. The BASE_120 genotypes were almost exclusively of race Mesoamerican origin, while including the Andean cultivars “Calima,” “Indeterminate Jamaica Red” (IJR), and “Quimbaya.” The seeding rate was 14 seeds per linear meter. The field trial received supplementary aerial irrigation to avoid drought stress. Weeds were controlled manually. Standard agronomic practices for weed and insect control were used in the trials.

No fertilizer was applied to the BASE_120 trials in order to produce soil conditions that favor nodulation and symbiotic N_2_ fixation. In the June 2015 trial, seeds were inoculated with a liquid suspension (10^7^ rhizobium cells per milliliter) of two strains of *Rhizobium*; *R. etli* (CIAT 632) and *R. tropici* (CIAT 899). The use of inoculant containing a mixture of more than one strain of *Rhizobium* is a recommended agronomic practice in common bean (Liu et al., 2020a,b). The inoculant was applied to the seed pre-planting using a sticker, PREMAX (1 ml/kg of seed), and the same CIAT 632 and CIAT 899 inoculant (100 g/kg of seed) by stirring the inoculant and the seed of each genotype in a container. The inoculum was also applied directly to the seeds in the row at planting, and to the base of the seedlings 1 week after planting, using a backpack sprayer. The inoculations for the second, third, and fourth growing seasons were performed using a peat-based inoculant.

Two plants per experimental unit were carefully dug up about 0.75 m from the end of the second row approximately 45 days after planting during flowering. Root nodule numbers were evaluated by assigning values from 1 to 9 using the CIAT scale (Schoonhoven and Pastor-Corrales, 1987), where 1 indicates that the root has >80 nodules, 3 = 41–80 nodules, 5 = 21–40 nodules, 7 = 10–20 nodules, and 9 = less than 10 nodules per plant. Seed samples from three replications of the trials conducted at the Isabela Substation in 2016 and 2018 were used to estimate %NDFA. Approximately five seeds of each sample were dried at 70°C until a constant weight was achieved then ground using a Wiley mini-mill (Thomas Scientific, Swedesboro, New Jersey, United States), and passed through a #40 mesh sieve, resulting in a fine powder. Samples containing 4.2 mg of the seed powder were packaged in 5 × 8 mm tin capsules (D1008, EA Consumables, Pennsauken, NJ, United States) and shipped to the University of California, Davis Stable Isotope Facility in 96-well plates. The ^15^N natural abundance method (Unkovich et al., 2008) was used to calculate %NDFA. The white bean R-99 was used as the non-N-fixing reference line. %NDFA was calculated as:

$$\%NDFA=\left[\frac{\left(\delta^{15}N_{R99}-\delta^{15}N_{BASE_{120_{Line}}}\right)}{\delta^{15}N_{R99}-\beta }\right]\times 100$$

where *β* represents the *δ*^15^N of the bean line grown under N-free conditions and relies on BNF for all N requirements. In this study, *β* was assigned the value of 0 because the R99 reference line does not nodulate. It was assumed that there was no isotopic fractionation in seed harvested from the genotypes grown under N-free conditions. The entries in the BASE_120 trail and the R99 reference bean line trial have similar patterns of phenological development and seed size.

Statistical analyses were performed for nodulation scores and %NDFA estimates using a GLIMMIX model from SAS/STAT14.1 (SAS Institute, Cary, NC, United States) to fit the model, estimate the main effects and interactions, and to compare the least squares (LS) means. Planting dates and bean genotypes were considered fixed effects, whereas replication within planting date was considered a random effect. A 95% probability level was used to establish statistical differences.

A genome-wide association study (GWAS) was performed using the phenotypic data for the BASE_120 genotypes, and their corresponding SNPs. The Base_120 was genotyped with SNPs (*n* = 2,05,293) generated from genotype-by-sequencing (GBS) reads of 469 Middle American genotypes (Oladzad et al., 2019). Only SNPs with minor allele frequencies ≥5% were extracted from this HapMap for the GWAS analysis using the GEMMA software (Zhou and Stephens, 2012, 2014). The kinship matrix, obtained from the centered relatedness algorithm, was included in the GWAS model as a random effect, and the structure matrix, obtained from the principal component analysis (PCA) using the Prcom function in R3.5, was considered a fixed effect (Price et al., 2006). The smallest number of principle components that accounted for at least 25% of the cumulative variation was used in the mixed GWAS model. Those SNPs whose values of *p* were in the lower 0.01 and 0.05% tail of the bootstrap distribution were considered significant. Bootstrapping was performed in R with 1,000 resampling. This approach is sensitive to the fact that the more genetic factors affecting a phenotype, the corresponding values of *p* would be higher. Artificial thresholds work best for traits in which a few genetic factors are involved (such as disease resistance loci). These will typically pass the conservative Bonferroni test. But for other traits, we believe the best way is to define the cut off value based on the trait and the specific population under study. That is why in the current study, 1,000 permutation tests and a *p* value bootstrap of two cutoff values were used. The amount of phenotypic variation explained by these significant SNPs was estimated by a likelihood-ratio-based (R_2_LR) analysis in R using the GenABEL package that included population structure and/or relatedness effects (Aulchenko et al., 2007; Sun et al., 2010). While GWAS results are indeed related to LD, it is important to note (and often little appreciated) that LD is a function of recombination. Genomic studies clearly show that recombination is not equal across the genome, especially as it relates to LD. For example, Moghaddam et al. (2016) clearly demonstrated LD must be considered in a regional context, and that regional context is population specific. The regional and population differences mean that LD values averaged across chromosomal regions, across whole chromosomes, and across populations have little value. What is important is the degree of LD in the narrow region, where we find the genetic factors associated with a specific phenotype. The question is what is the solution with regard to selecting a window size for candidate gene selection? The only true solution is to calculate the Mb per cM ratio around each genetic signal discovered by the GWAS analysis and choosing a specific cM distance to define the physical boundaries for candidate gene selection. The challenge though is that we do not know what genetic map to use since each biparental parent will have a different recombination frequency in the region of interest. Therefore, choosing a window size (admittedly somewhat arbitrarily), searching for a gene(s) in that region, and, based on previous analysis of the function of the gene as outlined in referred literature, makes a case for its possibility as a candidate gene. In the current study, candidate genes were identified within a conservative window size of ±50 kb interval of the significant SNPs. The possible effect of SNPs located inside a gene model or its promoter region was investigated using the SNPEff (Cingolani et al., 2012) database for common bean reference genome sequence version 2 (Oladzad et al., 2019). Finally, to check whether significant SNP markers near or within the major candidate genes associated with both traits, exhibit hitchhiking effects due to extensive LD, the pairwise LD in null model, using Tassel v5.0 (Bradbury et al., 2007), as well as in mixed model accounting for both kinship and population structure using R package LDcorSV (Mangin et al., 2012) were estimated.

## Results

The BNF capacity of a legume is associated with nodule-mediated nitrate (NO_3_^−^) production and general NO_3_^−^ uptake and mobilization. Integrating nodulation and %NDFA data with genotyping data in a genetic analysis enabled the identification of genomic intervals and candidate genes associated with BNF capacity and N metabolism under tropical soil conditions in common bean.

### Phenotypic Analyses of %NDFA Estimates and Nodulation Scores

Significant differences between seasons and among entries were found for %NDFA (Table 1). Although an overall season × entry interaction for %NDFA was not observed, several entries in the BASE_120 trial had significant F tests for the season × entry LS means slice (Supplementary Table S3). LS means for %NDFA from the June 2018 planting were generally lower than the LS means from the June 2016 planting. The range of LS means was comparable to %NDFA estimates for common bean in previous studies based on seed tissue (Polania et al., 2016). The BASE_120 control variety “ICA Pijao” had among the highest estimated %NDFA for both growing seasons.

**Table 1:** Type III tests of fixed effects for %NDFA and nodulation scores from bean abiotic stress evaluation (BASE) 120 field trials conducted at Isabela, Puerto Rico from 2016 to 2018.

			%NDFA			Nodulation score (1–9)		
Effect	Num DF	Den DF	*F* value	*Pr* > *F*	Num DF	Den DF	*F* value	*Pr* > *F*
Season	1	4	38.25	0.004	1	8	33.2	0.0004
Entry	111	426	1.61	5E-04	119	949	2.31	<0.0001
Season × entry	111	426	0.95	0.622	119	949	1.45	0.0022

Based on results from previous studies, Polania et al. (2016) estimated that the average %NDFA for common bean was 39%. Several BASE_120 lines including “Beníquez,” FBN 1205-31, FBN 1210-48, “INTA Centro Sur,” “Paraisito,” PR0443-151, PR1418-15′, “Rosetta,” “Sayaxché ML,” SB2-4, and SER 125 had LS means for %NDFA ≥ 50% in 2016 and %NDFA ≥ 40% in 2018 (Supplementary Table S3). In a N-free trial conducted by (Heilig et al., 2017), no significant differences in *δ*^15^N were found between flowering plants of “Pueblo 152,” “Zorro,” “Medalist,” and PR0443-151. Polania et al. (2016) used a constant *β* value for genotypes having type III growth habits based on greenhouse studies conducted by CIAT in Cali, Colombia. The use of a constant *β* value, however, did not change the ranks of %NDFA estimates among the entries. Unkovich et al. (2008) observed that the size of the *β* value is less important when %NDFA estimates are low (< 50%).

There were significant differences between seasons, entries, and season × entry interaction for nodulation scores. Nodulation and N_2_ fixation in common beans are sensitive to higher temperatures (Hungria and Franco, 1993) and soil moisture conditions (Hungria and Vargas, 2000; Polania et al., 2016), which may have contributed to variability in nodulation scores between seasons. The control variety “ICA Pijao” had among the highest nodulation scores across seasons. Other lines that combined superior %NDFA and a greater number of nodules included “Beníquez,” “INTA Centro Sur,” “Paraisito,” PR1418-15, and SB2-4. Among the 112 lines with data for both traits, the Pearson correlation between mean %NDFA and mean nodulation score was highly significant (*r* = 0.60). Diaz et al. (2017) reported that %NDFA was correlated with seed yield and suggested that enhanced BNF may be beneficial under abiotic stress.

### Association Mapping and Identification of Candidate Genes

**Figure 1:**
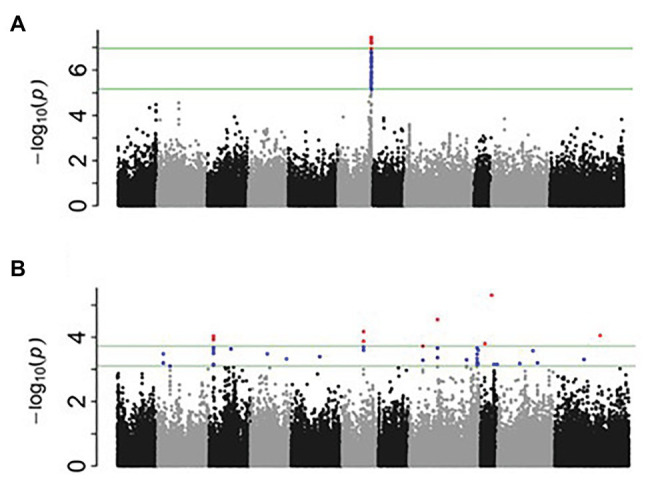
Manhattan plots generated from genome-wide association study (GWAS) analysis for nodulation **(A)** and percentage of N_2_-derived from atmosphere (%NDFA; **B**).

After filtering for MAF ≥ 0.05, a total of 1,25,745 SNPs out of 2,11,764 were extracted from the original HapMap (Oladzad et al., 2019). Two principal components explained 29.1% of the genetic variation and were used in the mixed linear model analysis. For nodulation score, a block of SNPs in high LD located on Pv06 between 28.19 and 28.25 in the 0.01% cutoff threshold were associated [log_10_(*P*) = 7.46] with the trait (Figure 1A). These SNPs explained 11% of the phenotypic variation. When SNPs in the 0.05% cutoff threshold were considered, the interval extended to 28.73 Mbp and cumulatively explained 13% of the phenotypic variation (Tables 2, 3). A total of 21 gene models were detected ±50 kb of this block. Four SNPs in complete LD at Pv06:28.198 Mbp; [−log_10_ (*P*) = 7.54] were located inside the gene model *Phvul.006G18000*, an ortholog of the Arabidopsis RAB GTPase. The auxin-responsive factor AUX/indole acetic auxin (IAA)-related gene model (*Phvul.006G181200*) was located 20 Kb upstream of RAB GTPase related gene model.

**Table 2:** GWAS results and *R*^2^ values for each trait.

Trait	Chr	Genomic interval (Mbp)	Peak SNP	−log_10_ (P)	*R*^2^	Cumulative *R*^2^ (*p* < 0.01)	Cumulative *R*^2^ (*p* < 0.05)
Nodulation	6	28.19–28.25	S06_28198455	7.46	0.11	0.11	0.13
%NDFA	9	25.89	S09_25892359	5.31	0.07	0.21	0.36
	8	28.42	S08_28424570	4.55	0.06		
	6	10.91	S06_10913425	4.18	0.07		
	11	32.89	S11_32896738	4.06	0.06		
	3	52.44	S03_5244212	4.04	0.06		
	9	6.00	S09_6000997	3.8	0.05		
	8	15.8	S08_15801341	3.72	0.06		

**Table 3:** Significant SNPs associated with nodulation in Middle American genotypes (redundant markers were removed).

Significance level^*^	Chr	SNP	Position (bp)	MAF	−log_10_(P)	Allele effect
*p* < 0.01	6	S06_28198455	28,198,455	0.252	7.46	−0.318
	6	S06_28203687	28,203,687	0.712	7.38	0.308
	6	S06_28212045	28,212,045	0.261	7.25	−0.306
	6	S06_28256196	28,256,196	0.297	7.18	−0.305
	6	S06_28142432	28,142,432	0.293	6.96	−0.319
*p* < 0.05	6	S06_28040985	28,040,985	0.703	6.82	0.303
	6	S06_28242723	28,242,723	0.275	6.80	−0.304
	6	S06_28260240	28,260,240	0.261	6.58	−0.298
	6	S06_28184429	28,184,429	0.694	6.55	0.300
	6	S06_28252919	28,252,919	0.297	6.44	−0.293
	6	S06_28153238	28,153,238	0.698	6.38	0.306
	6	S06_28045860	28,045,860	0.707	6.34	0.297
	6	S06_28249110	28,249,110	0.284	6.33	−0.300
	6	S06_28227691	28,227,691	0.703	6.20	0.292
	6	S06_28228447	28,228,447	0.293	6.19	−0.283
	6	S06_28087238	28,087,238	0.707	6.11	0.291
	6	S06_28257207	28,257,207	0.694	5.94	0.283
	6	S06_28077346	28,077,346	0.257	5.74	−0.298
	6	S06_27925696	27,925,696	0.716	5.57	0.290
	6	S06_28066399	28,066,399	0.306	5.49	−0.274
	6	S06_28022524	28,022,524	0.73	5.39	0.281
	6	S06_28273143	28,273,143	0.275	5.17	−0.263

For %NDFA, a total of 25 smaller effect, significant intervals containing 53 SNPs were detected in the 0.05% cutoff threshold (Figure 1B). The peak SNPs among these intervals cumulatively explained 36% of the phenotypic variation in %NDFA (Tables 2, 4). The peak SNP [−log_10_ (*P*) > 5.31] was detected at Pv09:25.89 Mbp and was located ~189 kb upstream of another annotated RAB GTPase gene. The second most significant SNP interval was 98 bp in length at Pv08:28.42 Mbp and was located ~2.5 kb upstream region of gene model *Phvul.008G157200*, an ortholog of a squamosa promoter binding protein-like 8 (*SPL8*). The SNPEff results revealed that this SNP was placed in the promotor regions of *SPL8* and potentially has a modifier effect. A SNP at Pv11:32.89 Mbp was detected 17 Mbp downstream of a small auxin up RNA (SAUR)-like auxin responsive protein family cluster. NO_3_^−^ transporters, *NRT1:7* and *NRT1.2* (orthologs to gene models *Phvul.002G067700* and *Phvul.002G067214*, respectively) flanked the Pv02:8.64 Mbp peak SNP within the heterochromatic region of Pv02. Finally, the Pv06:10.91 Mbp SNP interval is located ~40 kb downstream of a MATE efflux protein (gene model; *Phvul.006G028701*) known to be involved in flavonoid metabolism (Marinova et al., 2007), which is also critical for nodule development (Ng et al., 2015). Despite the importance of these candidate genes potentially involved in N metabolism, due to the quantitative nature of these traits, all the gene models within ±50 kb of the significant intervals were presented in the Supplementary Table S4. Furthermore, for more powerful trait discovery, single GWAS analyses were performed on phenotypic data from each season and year. The results and Manhattan plots are presented in the Supplementary Table S5 and Supplementary Figure S1. The results showed that the significant SNPs on Pv06 for nodulation which was detected from Ls-mean values within a LD block, was also identified in 3 seasons/years (November 2015, June 2018, and June 2015) and interestingly the significant SNP on Pv02; 14.33Mbp was identified in both nodulation and %NDFA in June 2016 and June 2018, respectively. This SNP was upstream of gene model *Phvul.002G088100*. Furthermore, the most significant SNPs on Pv09, Pv08, Pv06, and Pv11 that were detected from Ls-mean values for %NDFA, were also identified in the single GWAS analysis of June 2016 and June 2018. The pairwise LD values were reported as squared allele frequency correlation in the Supplementary Table S6 for both null model and mixed model accounting for both population structure and relatedness. Based on these values, the significant SNPs are not in high LD specifically when population structure and relatedness are accounted in the model. However, some degree of LD was observed between Pv08:28.42 and two other SNPs on Pv02:8.64 and Pv09:25.89Mbp in the null model and may very well be due to the gene by gene interactions within this pathway. As a final point, for each peak SNP, the allele associated with higher %NDFA and lower nodulation scores (higher number of nodules) were determined, and the mean of each trait of all possible haplotypes was calculated (Table 5). Selecting the genotypes for negative alleles for nodulation and positive alleles for %NDFA revealed that although both nodulation and %NDFA are indicators of BNF capacity, different genetic factors control each of these traits, and selection based on marker data should be independent for each trait (Supplementary Table S7).

**Table 4:** Significant SNPs associated with %NDFA in Middle American genotypes (redundant markers were removed).

Significance level	Chr	SNP	Position (bp)	MAF	−log_10_(*p* value)	Allele effect
*p* < 0.01	9	S09_25892359	25,892,359	0.156	5.32	7.461
	8	S08_28424570	28,424,570	0.486	4.55	−12.486
	6	S06_10913425	10,913,425	0.514	4.18	−10.089
	11	S11_32896738	32,896,738	0.401	4.06	9.660
	3	S03_5244212	5,244,212	0.137	4.04	8.347
	9	S09_6000997	6,000,997	0.896	3.80	−9.593
	8	S08_15801341	15,801,341	0.491	3.73	−10.960
	6	S06_10913399	10,913,399	0.505	3.71	−8.890
	3	S03_23219229	23,219,229	0.052	3.64	−17.289
	8	S08_62446399	62,446,399	0.33	3.60	−4.535
*p* < 0.05	10	S10_24164888	24,164,888	0.052	3.58	−13.561
	2	S02_8646280	8,646,280	0.344	3.48	6.645
	8	S08_61984614	61,984,614	0.425	3.47	6.321
	5	S05_19573422	19,573,422	0.269	3.40	−5.613
	8	S08_61970086	61,970,086	0.401	3.34	−9.526
	4	S04_42699708	42,699,708	0.377	3.33	−7.919
	11	S11_26070577	26,070,577	0.448	3.31	6.932
	8	S08_51857313	51,857,313	0.061	3.30	−12.146
	8	S08_61995491	61,995,491	0.184	3.24	−5.116
	10	S10_27861921	27,861,921	0.476	3.20	6.583
	10	S10_13747196	13,747,196	0.061	3.19	10.334
	8	S08_62397050	62,397,050	0.16	3.18	−7.471
	9	S09_34108296	34,108,296	0.127	3.16	6.586
	9	S09_37907474	37,907,474	0.344	3.16	−6.651
	2	S02_14339482	14,339,482	0.193	3.10	7.117

**Table 5:** Efficiency of the selected markers.

Trait	SNP locus	Allele	Mean positive allele	Mean negative allele	*p* value
Nodulation	S06_28203687	C/G	6.84	7.37	3.46E-08
Trait	SNP locus	Allele	Mean negative allele	Mean positive allele	*p* value
% NDFA	S09_25892359	C/T	38.03	49.78	4.83E-06
	S08_28424570	C/G	33.08	50.88	2.81E-05
	S06_10913425	C/T	31.15	51.81	6.62E-05
	S02_8646280	G/T	36.91	40.32	3.30E-04

## Discussion

The contribution of BNF to common bean growth and development is key to its performance as a field crop, especially under field stress conditions. While it has been demonstrated previously that degree of nodulation and %NDFA are two key phenotypes related to BNF, the underlying genetic factors that control these traits were not studied before. In this study, a combination of detailed phenotypic data of a population with modern breeding alleles and in-depth genotyping of that population enabled the discovery of key genetic factors for nodulation and %NDFA. The individual genetic analysis for each trait was robust. These genetic analyses also enabled a description of the interactions of a genetic pathway that controls BNF.

The phenotypic analysis confirmed that the range of LS means for seed %NDFA in this common bean population was comparable to previous studies based on seed tissue (Polania et al., 2016). While nodulation and N_2_ fixation in common beans are sensitive to high temperatures (Hungria and Franco, 1993) and inadequate soil moisture (Hungria and Vargas, 2000; Polania et al., 2016) two conditions that affected the variability in nodulation phenotypes/scores in this study, the consistency of the range of phenotypic when compared with previous studies suggests the data was representative of the modern germplasm and appropriate for performance and genetic analysis. This study identified bean lines capable of obtaining a significant portion of the N needed for growth and development from BNF. The BASE_120 trials were conducted in soils at Isabela, Puerto Rico that have low levels N and P. Therefore, these results should be relevant to small-scale bean producers in the tropics, who often do not have access to fertilizer inputs. Moreover, Heilig et al. (2017) evaluated %NDFA in a RIL population derived from the cross “Puebla 152 x Zorro” (Mesoamerican) at Isabela, Puerto Rico during 2012 and 2013. This study was conducted under conditions similar to the BASE 120 field trials in our study; an unfertilized oxisol (Coto Clay). Significant differences were observed among RILs for %NDFA. Although narrow sense heritability was not estimated, the significant difference among RILs suggests that additive genetic effects were important in the expression of %NDFA in this “Puebla x 152 x Zorro” RIL population.

Associating genetic variation in BNF with specific genomic regions and SNPs offers an important tool for molecular breeding. In this study, we utilized the 200 k SNPs generated previously from a USAID bean project (Oladzad et al., 2019) and identified genomic regions associated with two critical phenotypes associated with BNF in the Middle American gene pool. Within these regions, six important candidate genes previously determined by cellular and physiological analysis of transgenic plants to be associated with N_2_ fixation (Borg et al., 1997; Jürgens, 2004; Wang, 2004; Blanco et al., 2009; Bloch et al., 2011) were identified. The major SNP for nodulation is located in a gene encoding a small Rab GTPase protein. These classes of proteins were shown to have a positive regulatory role in root hair tip growth (Borg et al., 1997; Meschini et al., 2008; Bloch et al., 2011), a developmental function necessary for the establishment of the bean/rhizobia symbiosis. Gene expression analysis suggested a common bean Rab GTPase protein regulates the intercellular vesicle trafficking in root, which determines cell polarity in growing root hairs (Jürgens, 2004; Wang, 2004; Blanco et al., 2009), and therefore is required for nodulation. Silencing this gene reduced or eliminated nodulation by rhizobia (Blanco et al., 2009). A parallel molecular network involving Rab GTAPase family proteins, reactive oxygen species (*ROS*), phosphatidylinositide, and Ca^+2^ signaling pathways establishes the symbiotic interactions when legumes are infected by rhizobacteria (Jürgens, 2004; Wang, 2004; Peleg-Grossman et al., 2007).

The AUX/IAA-related auxin-responsive factor located 20 kb upstream of the Rab GTPase gene is another candidate. Shoot-to-root auxin transport regulates nodule number. Auxin transport was shown to be closely associated with nodule development using biological function, gene expression, and high performance liquid chromatography-immunoassay analyses (Atzorn et al., 1988; Pii et al., 2007; Mathesius, 2008; Barraza et al., 2018; Zou et al., 2019). Upon rhizobia infection, an alteration of the auxin balance is a prerequisite for nodulation (Mathesius, 2008; Jin et al., 2012; Kohlen et al., 2018). Van Noorden et al. (2016) showed that under N_2_-rich conditions, the number of nodules is reduced because NO_3_^−^ inhibits the synthesis of flavonoids in the host root (Coronado et al., 1995). Because flavonoids control the accumulation of auxin at the site of nodule formation (Ng et al., 2015), auxin transport consequently the number of nodules can be affected by the presence of NO_3_^−^. However, Van Noorden et al. (2016) determined that *IAA* accumulation at the root-shoot junction of *Medicago truncatula* was not affected by NO_3_^−^ 24 h after inoculation. According to Liu and Murray (2016), the flavonoids are the key molecules for biosynthesis of nod factors (NFs), which are the signaling molecules produced by rhizobacteria during interaction with plant. However, it has been shown that flavonoids can also simulate nod expressions even before rhizobia-legume interaction through rhizobial chemotaxis (Hirsch et al., 2001).

Data of %NDFA are used as a proxy for “N^15^ natural abundance” to quantify the contribution of N_2_ fixation in common bean to N_2_ metabolism. The GWAS results of that data identified two candidate genes related to nodulation, *SPL8*, and SAUR-like auxin responsive protein, and two candidate genes were related to NO_3_^−^ transport, *NRT1:2* and *NRT1.7*. The *SPL* gene family is a target gene for miR156 (Yu et al., 2015), which affects the regulation of nodule development in plants (Wang and Wang, 2015). Overexpression of this miRNA downregulates nodulation in legumes by silencing downstream *SPL* genes. In both common bean and soybean, it has been shown that SPL activates the transcription of miR172, which is highly accumulated in the mature nodules. miR172 binds APETALA2 (AP2), protein positively nodule senescence genes in common bean. In mature nodules, AP2-1 is silenced by miR172 (Íñiguez et al., 2015; Nova-Franco et al., 2015). In soybean, it has been demonstrated that AP2-2 controls the non-symbiotic hemoglobin expression (Hb1), which affects the level of nodulation by regulating nitrogenase activity (Yan et al., 2013). However, it is not clear which subfamily of SPLs in this regulatory network is associated with nodulation in common bean. *SAURs* were also detected as potential candidate genes. These are the largest families of early auxin response genes (Ren and Gray, 2015), and auxin families are associated with root nodule symbiosis.

Nitrate transporters *NRT1:2* and *NRT1.7* are both members of the *NRT1* NO_3_^−^ transporter subfamily system (later renamed the NPF family Léran et al., 2014) that transfer a wide range of molecules including auxin and nitrates (Fan et al., 2009). The NPF family uses a proton gradient to transport NO_3_^−^ from the soil to different parts of the plant (Segonzac et al., 2007). The *NRT1:2* mRNA was found in root hairs and in the epidermis of young and mature roots (Huang et al., 1999; Guo et al., 2002), while *NRT1.7* mRNA was located in the phloem tissue of older leaves. This suggests a role of NRT1.7 in the remobilization of NO_3_^−^ from source to sink. This role was further confirmed by analyzing the N^15^ content of the older and younger leaves in wild (*NRT1.7*) and mutant (*nrt1.7*) Arabidopsis (Fan et al., 2009). In wild type genotypes, N^15^ was remobilized from older leaves to the younger leaves, while in the mutant; higher amount of NO_3_^−^ was accumulated in older leaves and was not transferred to the younger tissues.

**Figure 2:**
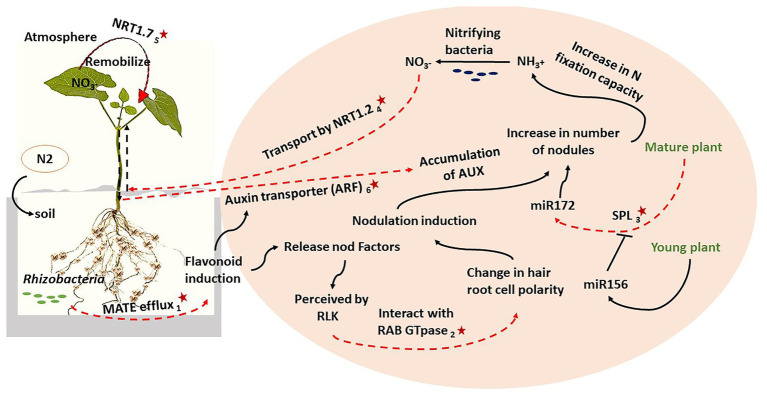
The key candidate genes (red stars) from both traits were integrated with previous results on nitrogen (N_2_) fixation to describe a biological nitrogen (N_2_) fixation (BNF) pathway: (1) Phvul.006G028701, (2) Phvul.006G18000, (3) Phvul.008G157200, (4) Phvul.002G067700, (5) Phvul.002G067214, and (6) Phvul.006G181200.

Integrating the information from both nodulation score and %NDFA shows that the SNPs which mapped in or adjacent to candidate genes are associated with BNF in common bean. Figure 2 integrates our genetic results into a N_2_ metabolism pathway based on previous research to show how the candidate genes interact to provide a high level of N_2_ fixation in low fertile soil. For this genotype, rhizobacteria infection triggers the expression of the MATE efflux gene involves in flavonoid metabolism. Flavonoids prompt the transcription of genes related to the biosynthesis of the NFs in the soil which are perceived by the LRR domain of plasma membrane-localized receptor-like kinases (RLKs; Ferguson et al., 2010; Kamfwa et al., 2015). This complex would interact with a RAB family GTpase (Choudhury and Pandey, 2016), which alters the hair root cell polarity, resulting in nodule induction. The expression of auxin transporters from shoot to root also triggers flavonoids release, which would also lead to high auxin accumulation in the root resulting in greater lateral root development and consequently greater nodule number. Moreover, upon Rhizobia infection, the expression of SPL would increase miR172 levels until nodulation reaches it maximum accumulation in the mature plant. Higher levels of miR172 also improve root growth which subsequently increases the rhizobia infection and therefore a higher expression of nodulation regulatory-related genes. This leads to further nodule as the plant matures (Nova-Franco et al., 2015). The overall effect of this integrated genetic pathway is an increase in N fixation capacity. The ammonium (NH_3_^+^) produced in this process can be converted to NO_3_^−^ by nitrifying bacteria. The NO_3_^−^ will then be transported from root to shoot by *NRT1.2*, and later, remobilized by *NRT1.7* from older leaves to the younger leaves. During plant maturation, NO_3_^−^ will be distributed to the seeds which increases %NDFA (Diaz et al., 2017). As a final point, it should not be rolled out that despite the statistical power of GWAS discovery of the candidate genes for complex traits, still there is potential for missing medium and low-effect associations specifically after filtering for allele frequency which is commonly employed in GWAS analysis. It has been suggested that sequencing the candidate genes specifically in individuals with extreme phenotypes of a quantitative traits may be able to detect other associations but the sample size at the extremes of a quantitative trait is also debatable because it can be affected by small odds ratios (Manolio et al., 2009). However, using GWAS to introduce a breeder “friendly” marker was beyond the scope of this study, rather it aimed using GWAS as a genetic approach to understand the molecular pathways.

## Data Availability Statement

The original contributions presented in the study are included in the article/supplementary material, further inquiries can be directed to the corresponding authors.

## Author Contributions

AO, AG, RM, and CJ performed data analysis and JB, TP, and PM supervised the data analysis. JB and TP designed the phenotypic experiment and AO and PM designed the genomics approach for the association mapping. AO, AG, and RM discussed the results and the interpretation of the final data and JB, TP, and PM provided suggestions to improve it. All authors contributed to the article and approved the submitted version.

### Conflict of Interest

The authors declare that the research was conducted in the absence of any commercial or financial relationships that could be construed as a potential conflict of interest.
