# Supplementary material for: Genetic Factors Associated With Nodulation and Nitrogen Derived From Atmosphere in a Middle American Common Bean Panel
Source: Front Plant Sci. 2020 Dec 15;11:576078. doi: 10.3389/fpls.2020.576078 (PMC7769817; doi:10.3389/fpls.2020.576078)
Supplement: Supplementary file 2 [file Presentation_1.pptx]

## Slide 1
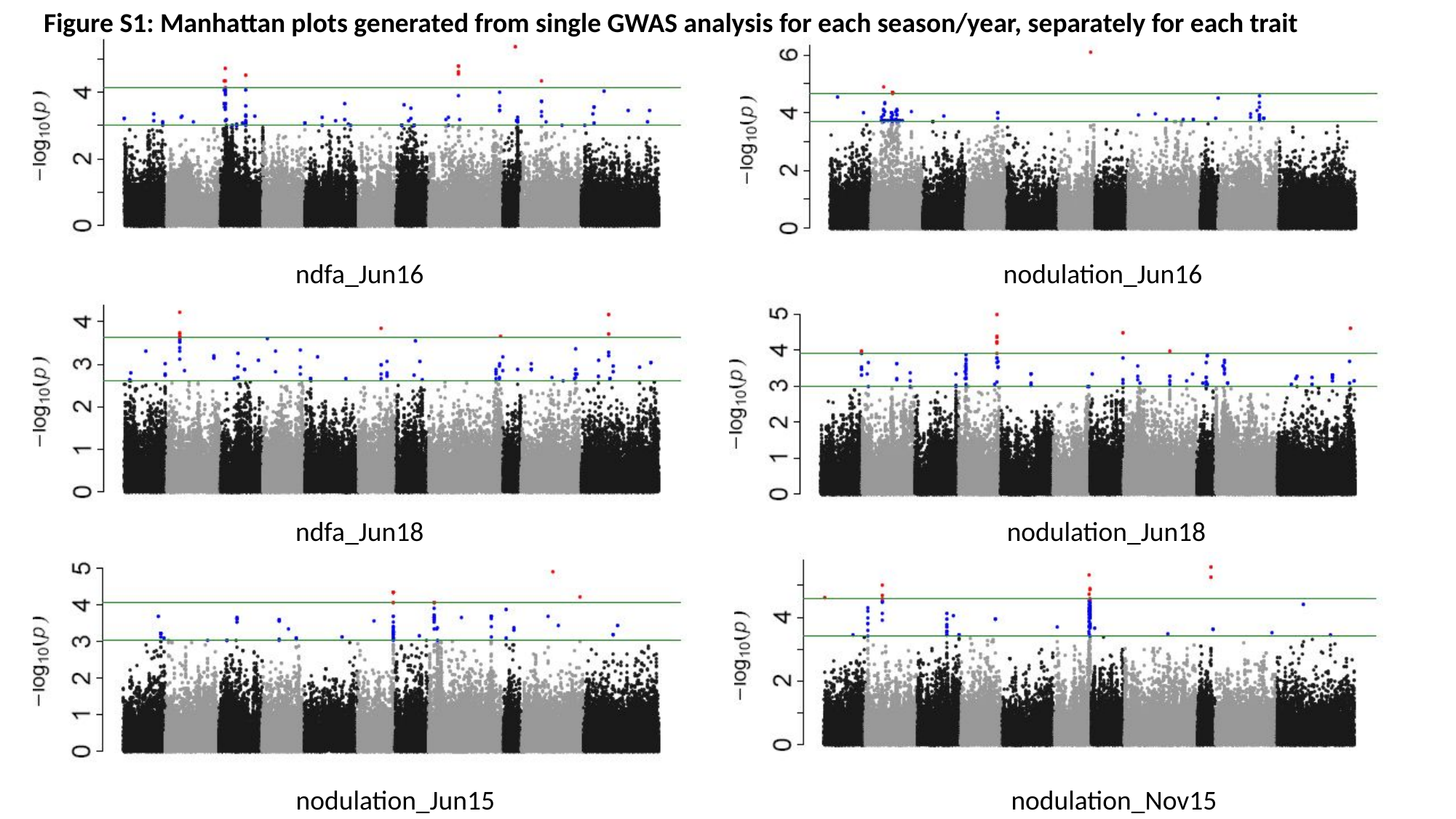

Figure S1: Manhattan plots generated from single GWAS analysis for each season/year, separately for each trait
ndfa_Jun16
nodulation_Jun16
nodulation_Jun18
ndfa_Jun18
nodulation_Jun15
nodulation_Nov15
